# Battery Testing and Discharge Model Validation for Electric Unmanned Aerial Vehicles (UAV)

**DOI:** 10.3390/s23156937

**Published:** 2023-08-04

**Authors:** Attilio Di Nisio, Giulio Avanzini, Daniel Lotano, Donato Stigliano, Anna M. L. Lanzolla

**Affiliations:** 1 Department of Electrical and Information Engineering, Polytechnic University of Bari, Via E. Orabona 4, 70125 Bari, Italy; 2 Department of Engineering for Innovation, Università del Salento, Via per Monteroni, 73100 Lecce, Italy

**Keywords:** battery, lithium polymer battery, discharge model, UAV, power, capacity

## Abstract

Electrical engines are becoming more common than thermal ones. Therefore, there is an increasing interest in the characterization of batteries and in measuring their state of charge, as an overestimation would cause the vehicle to run out of energy and an underestimation means that the vehicle is running in suboptimal conditions. This is of paramount importance for flying vehicles, as their endurance decreases with the increase in weight. This work aims at finding a novel empirical model for the discharge curve of an arbitrary number of battery pack cells, that uses as few tunable parameters as possible and hence is easy to adapt for every single battery pack needed by the operator. A suitable measurement setup for battery tests, which includes voltage and current sensors, has been developed and described. Tests are performed on both constant and variable power loads to investigate different real-world scenarios that are easy to reproduce. The main achievement of this novel model is indeed the ability to predict discharges at variable power based on a preliminary characterization performed at constant power. This leads to the possibility of rapidly tuning the model for each battery with promising accuracy. The results will show that the predicted discharged capacities of the model have a normalized error below 0.7%.

## 1. Introduction

Electrical propulsion is becoming more important. Internal combustion motors are usually bulky and heavy when compared to electric ones since they show low efficiency due to thermal losses that need to be addressed, for example, with specific combustion patterns [1]. Other important aspects when comparing combustion engines and electric engines are the pollutant emissions, lower for electric vehicles [2], and the noise of the engine itself [3]. Moreover, there is the need for external systems, such as injectors or compressors, and internal combustion motors require monitoring of fuel and oil parameters, e.g., level, quality, and contamination [4,5,6,7,8]. Thanks to the improvement of DC and AC motor performance, innovative and more efficient power management and storage techniques, and increase in power and energy density of battery packs [9,10,11,12], many different classes of ground, aerial and underwater vehicles are being designed with a fully electrical powertrain, such as Unmanned Aerial Vehicles (UAV)s [13,14,15,16], cars [17,18,19,20], mobile robots in diverse industrial applications [21,22,23,24] and even short-range aircraft [25]. Moreover, the extensive use of distributed sensors allows for unprecedented levels of autonomy in mission management and totally novel (and sometimes disruptive) human–machine interfaces. In most cases, batteries still pose the most relevant technological bottleneck for the full-scale development of these classes of electrically powered vehicles. Hence, there is an increasing interest not only in battery performance but also in their correct characterization.

When powertrains based on thermal engines are considered, it is easy to predict and measure almost exactly fuel flow, overall fuel consumption, and residual fuel level in the tanks of the vehicle (as an example, the fuel required for a given flight distance at a given cruise speed). When electric engines are adopted, estimating the actual effective capacity and state of charge of batteries is far from easy due to many factors, such as the effect of the electric load, operational conditions, and aging. On one side, the qualitative features of the discharge process are well known: for a given battery pack with a nominal capacity, higher discharge currents result in a reduction in the effective charge; battery voltage decreases almost linearly with time, for a constant current discharge process, with a sudden decrease when the battery is close to fully discharged; the effective battery capacity is also reduced by an aging process, after several charge/discharge cycles; environmental conditions (such as temperature) may also affect the effective charge available from a battery. All these phenomena become even less predictable when variable electrical loads and/or incomplete charge/discharge processes are considered.

Nonetheless, it is clear that the safe use of an electrically powered vehicles clearly requires that the amount of energy stored in the battery pack is sufficient at every time instant for completing the expected task, possibly with an adequate power reserve in case of emergencies or unexpected changes in mission requirements. If the state of charge of the battery is overestimated during a mission, the risk of running out of energy before the mission is complete poses a serious risk. However, similarly, if the state of charge is underestimated, the use of the vehicle may become largely suboptimal. The estimate of the effective capacity of batteries during vehicle operations is also relevant for the design phase. The sizing of the vehicle, and within this procedure sizing of the battery pack, clearly relies on an estimate of the discharge process during the sizing mission. This aspect is important for all vehicles, but it is particularly relevant for electrically powered rotary-wing aircraft, where the power required at hovering and at low speed grows superlinearly with weight, which implies that, beyond a certain fraction of total takeoff weight, increasing battery weight would cause endurance to decrease [26].

Even other small integrated devices can benefit from a reliable estimate of the battery state of charge. Examples are implantable medical devices and equipment used for health monitoring of a patient’s heartbeat and pressure throughout the day, where the capability of exactly estimating battery duration would allow for the use of standalone devices [27,28,29,30]. Hence, the definition of a reliable discharge model with the objective of estimating the residual battery charge represents a result relevant in applicative fields well beyond those represented by ground and aerial vehicles.

The focus of the research is on Lithium-Polymer (LiPo from now on) battery packs, which are the most used batteries in electrically powered vehicle applications. Various operational conditions are considered. As a contribution with respect to previous works, where mathematical models and the corresponding set of parameters were determined for standalone LiPo battery cells [31,32,33], this paper presents a novel approach to finding a model suitable for LiPo battery packs composed by an arbitrary number of cells in series. More in detail, the objective of the paper is twofold. An experimental setup is presented first, which can reproduce arbitrary load cycles, for diverse applications and/or missions of electrically powered vehicles. A test campaign is then performed, which is aimed at testing an existing empirical discharge model [34] and extending its validity.

In this respect, the type of electrical load is relevant. In many applications of interest, a constant power discharge process is representative of the actual battery load, such as during the cruise of a fixed-wing aircraft or a hover condition of a multirotor vehicle or electrically powered helicopter. In such a case, an optimized design strategy based on an empirical discharge model was proposed in [34]. In the present study, not only constant power but also two power step tests were performed experimentally for verifying the model. Then, with the aim of generalizing that model for more complex discharge processes, a variable power consumption was considered, performing tests with power profiles described by Amplitude Modulated Pseudo-Random Bit Sequences (APRBS). A comparison of all the tests with different parameters was carried out. The main used metric was the RMSE between the measured discharged capacity and the one predicted by the model. An evaluation of the final error at the end of discharge was also calculated. The fitted model proved to be reliable, with little error with respect to the sampled values. The experiments are easy to reproduce so that multiple commercially available batteries can be tested. It is noteworthy that the extension to variable loads allows for the use of the considered battery discharge model in a much wider range of applications, including autonomous cars and/or rovers, which often work with a highly variable electrical load, due to different operational and environmental constraints.

The novel model presented in this work could complement many others that recently appeared in the literature. For instance, many researchers are working on estimating the State of Health (SOH) of Li-Ion batteries. This application usually relies on Long-Short Term Memory Neural Networks (LSTM) [35,36] that require lots of data for training and will not estimate the discharge during the operation. Combining both methods can lead to the evaluation of both the life span of the battery and its usage during the operation. Similar research is being carried out for different battery technologies and chemistry and using different techniques, such as the ones presented in [37,38] for Li-S and Zn Hybrid-Ion batteries.

This paper is structured as follows. A concise summary of the theoretical models on the basis of the definition of the electrical load will be given in Section 2, to provide some understanding of how the model could be used for estimating battery duration in a realistic operational scenario for aerial vehicles. In Section 3, experimental setup and methods are presented, including preliminary operations for finding internal resistance and the discharge procedure using a programmable electronic load. The definition of test conditions and model parameter estimation are reported in Section 4. Experimental results and conclusions are reported in the last sections of the paper.

## 2. Theoretical Models of Power Requirement

For any electrically powered vehicle, the battery must supply a total power equal to

(1) $$P_{tot}=P_{S}+P_{R},$$

where $P_{S}$ is the power required by on-board systems (including the payload, if not equipped with an independent power source), and $P_{R}$ is the power required for motion. Different expressions can be derived for $P_{R}$ in the case of fixed-wing aircraft, rotary-wing aircraft, and ground vehicles. Due to the variety of applications and power requirements, from almost stationary to rapidly changing, it will be clear that the discharge model should be tested against different power profiles.

### 2.1. Fixed-Wing Aircraft

In this case, $P_{R}$ is the power required for flight, which can be divided into three contributions,

(2) $$P_{R}=DV+W\dot{h}+\frac{W}{g}V\dot{V},$$

which represent, respectively, the power dissipated by drag *D*, the power required to climb at a climb rate $\dot{h}$, and power used for accelerating the aircraft, for which the weight is *W*. When flying at velocity *V* and at an altitude *h*, where the air density is ρ, airplane drag is equal to [39]

(3) $$D=½\rho V2S\;C_{D},$$

where *S* is the reference wing planform area. The drag coefficient $C_{D}$ can be modeled by means of a parabolic drag polar,

(4) $$C_{D}=C_{D0}+K\;C_{L}^{2},$$

where the parasite drag coefficient $C_{D0}$ and the induced drag factor *K* are constant when flying at subsonic speed, as is the case for all electrically powered airplanes. Upon substitution of the lift coefficient

(5) $$C_{L}=\frac{W}{\frac{1}{2}\rho V^{2}S},$$

into the expression of the drag polar, the expression for $P_{R}$ achieves the form

(6) $$P_{R}=AV^{3}+\frac{B}{V}+W\dot{h},$$

with $A=½\;\rho \;S\;C_{D0}$ and $B=2\;K\;W^{2}/(\rho \;S)$, where the contribution due to acceleration is dropped assuming that the duration of acceleration transients is short compared to mission time, thus making its contribution negligible within the overall energy balance of a whole mission.

### 2.2. Rotary-Wing Aircraft

In the case of rotary-wing aircraft, the lifting force is obtained by the rotation of one large rotor, in the case of a conventional single-main rotor helicopter configuration, or the rotation of pairs of counter-rotating rotors in the case of multirotor configurations, as in the case of the widely used adopter quad-rotor configuration, typical of many electrically powered small-size drones. This means that also the lifting force is obtained at the expense of shaft power, delivered by the vehicle engine(s).

For a conventional helicopter configuration, the contributions to the required power $P_{R}$ are given by the sum of power dissipated by the fuselage, power required by main and tail rotors, and power required to climb, that is,

(7) $$P_{R}=D_{fus}V+P_{MR}+P_{TR}+W\dot{h},$$

where power associated with the variation of vehicle kinetic energy is again neglected, as in the case of fixed-wing aircraft.

Equation (4) can be simplified for a multirotor, i.e., a quadcopter, moving in a straight line at a slow speed:

(8) $$P_{R}=D_{fus}V+P_{S}+P_{h}+W\dot{h}\approx P_{h}+W\dot{h},$$

where $D_{fus}V$ and the power for the on-board systems $P_{S}$ are negligible with respect to the power requested by the motors for holding the altitude ($P_{h}$) and the power required to climb ($W\dot{h}$), for what concerns this research.

The mechanical power requested by the motors can be expressed at first by a sufficiently reliable model based upon the disk actuator theory, momentum balance, and blade elementary theory [40] as:

(9) $$P_{R}=C_{P}\rho \Omega^{3}D^{5},$$

where Ω is the rotation speed expressed as revolutions per second and D is the propeller diameter. The same applies to the thrust produced by the spinning propeller:

(10) $$T=C_{T}\rho \Omega^{2}D^{4}.$$

The power and thrust coefficient $C_{P}$ and $C_{T}$, respectively, are given by the propeller manufacturer.

## 3. Experimental Setup and Methods

The target of the work is to accomplish different tests to estimate the discharged battery capacity and compare it to the reference values calculated by numerical integration of the sampled current over time. 

### 3.1. Preliminary Operations: Internal Resistance Measurements

The internal resistance is an important parameter for monitoring battery discharge since it allows the estimation of the open circuit voltage of the battery and safely stops discharge when its value drops below a safety threshold. For this reason, resistance is the first parameter that was evaluated on the battery pack used throughout the tests.

Among the various methods available for estimating the battery’s internal resistance, one of the most used procedures is the VDA Current Step Method. With this method, the battery is stimulated with a discharge current pulse at 20 C current (equal to 20 times the current that discharges the nominal capacity in one hour) for 18 s and the internal discharging resistance value is evaluated at 2 s, 10 s, and 18 s since the discharge pulse is applied. It is also possible to estimate the internal charging resistance by applying a charge current pulse in a similar way.

**Figure 1:**
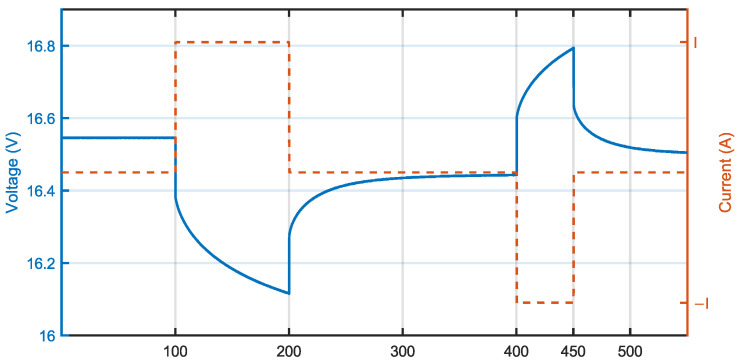
Measurement of internal resistance according to amended VDA test procedure (example at 8 A charge/discharge current). Positive current is discharge current.

Due to the limitation of the instrumentation used in the laboratory, the above-mentioned procedure was amended to satisfy the constraints of the equipment. In particular, the current was limited up to 8 A, the discharge pulse had a duration of 100 s and the charge pulse had a duration of 50 s. The applied method is graphically described in Figure 1.

**Figure 2:**
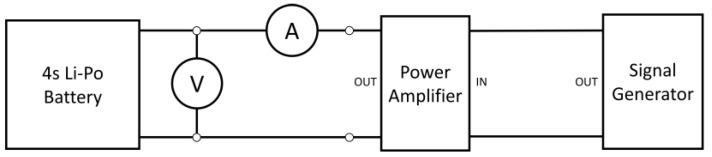
Setup for measurement of internal resistance.

The experimental setup used to evaluate internal resistance consisted of two multimeters, a signal generator, and a power amplifier connected as shown in Figure 2.

The Keysight DSOX1204G oscilloscope with a built-in signal generator was used to generate the discharge and charge control signal to be input to the Toellner TOE 7621 four-quadrant power amplifier.

To evaluate the internal resistance of the battery, several experiments were performed by applying discharge and charge currents of 2 A, 4 A, 6 A, and 8 A. The details of the test are shown in Table 1.

The VDA procedure calculates the battery’s internal resistance as the ratio between the voltage variation and the current variation at the instant of interruption of the discharge pulse. In other words, it is possible to calculate the internal resistance as the ratio between the amplitude of the voltage drop and the amplitude of the current pulse at the end of the discharge:

(11) $$R_{int}={\left.\frac{\Delta V}{\Delta I}\right|}_{t_{off}}.$$

**Figure 3:**
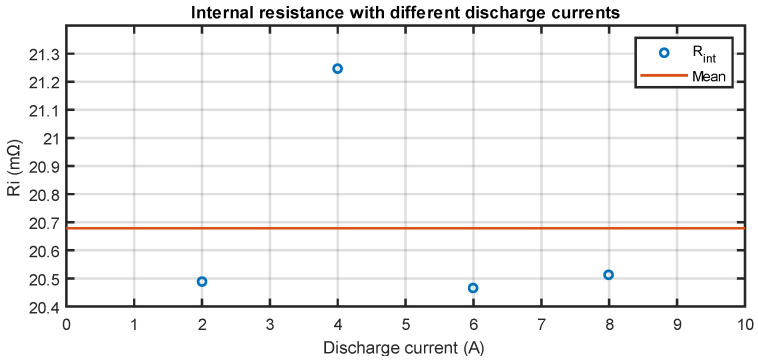
Calculated internal resistances.

The internal resistance values calculated from the tests are reported in Table 2. Given that the internal resistance values obtained for the various discharge currents differ from each other by less than 1 mΩ, the hypothesis of a constant resistance model for different test currents is considered. The constant value calculated by the model, that is the mean value, is equal to 20.68 mΩ with a 95% confidence interval between 20.07 mΩ and 21.29 mΩ under the Gaussian distribution hypothesis. The root mean square error is equal to 0.38 mΩ. The plot in Figure 3 shows the distribution of the calculated resistances and the fitted model.

### 3.2. Discharge Tests Setup and Procedure

**Figure 4:**
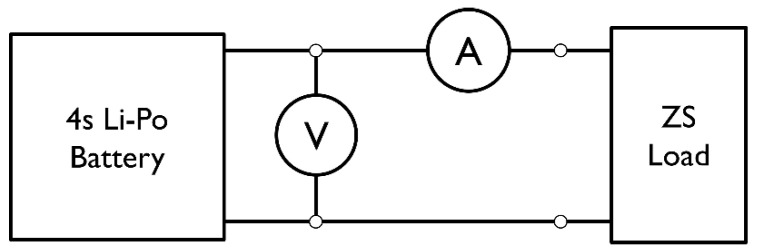
Setup for discharge test.

To evaluate the battery State of Charge (SOC) and characteristic parameters, the measurement setup and the instruments described below are used. Furthermore, the circuit diagram is schematized in the following Figure 4.

The lithium polymer battery under test is a Multistar 10.0 with a nominal capacity of 10 Ah and a nominal constant discharge rate of $C_{rating}=$ 10 $h^{-1}$, so that the battery can output nominally:

(12) $$I_{max}=10\;Ah\cdot C_{rating}=100\;A.$$

It is a battery pack made of 4 Li-Po cells put in series (4S1P), hence its nominal voltage is 14.8 V.

An ammeter for current measurement is connected in series to this battery and a multimeter for voltage measurement is connected through the use of a different wiring, with the aim of avoiding the well-known potential drop at the passage of the high currents, which changes due to the increasing resistance with the heating of the cables.

For this application, two GW Instek GDM-8351 multimeters are used, configured with a range of 100 V for voltage measurements and 10 A for current measurements, respectively. 

The ZS506-4 programmable electronic load is used to discharge the battery. To perform the power control the two multimeters are setup in a four-wire configuration for the measurement of voltage and current.

**Figure 5:**
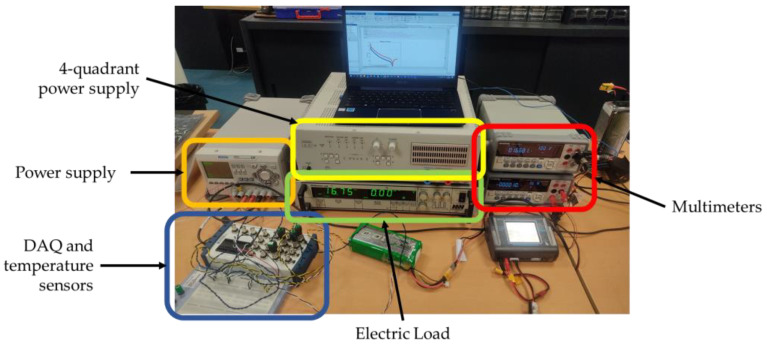
Instruments used in the setup for the discharge tests.

The experimental setup is showed in Figure 5.

The automatic control of the instruments and measurements acquisition is carried out using the VISA library; for the synchronization of the measurements operations, suitable timers are used, which allows us to acquire voltage and current measurement at a reading rate of 1 sample/s, associating a time value to each pair of measurements from which it is possible to carry out the post-processing described below.

Before activating the electronic load and proceeding with the battery discharge, 100 samples are acquired by means of multimeters, in order to measure the initial voltage and evaluate the current offset error.

Experiments described in the following subsection have been designed by assuming a capacity $C_{0}$ corresponding to a safety depth of discharge at the end of the experiment of about 55% of 10 Ah nominal capacity, after a preliminary assessment of battery behavior in which the minimum voltage of 3.5 V/cell was reached in some experiments. Indeed, in each test, the discharge process was monitored and then interrupted early in case the measured voltage, added to the voltage drop due to the internal resistance, becomes equal to or lower than the nominal discharged-state voltage of the battery to avoid its damage. The internal resistance values are evaluated before carrying out the tests, as explained in Section 3.1.

### 3.3. Model Estimation

The discharged capacity up to time t starting from time zero is denoted as C and is defined by:

(13) $$C\left(t\right)=\int_{0}^{t}I\left(t\right)\cdot dt,$$

where I is the measured current. As well known in the literature, the voltage of the battery decreases during usage, hence the current increments during this time in order to make constant P=V·I.

An estimation $\hat{C}\left(t\right)$ of the discharged capacity $C\left(t\right)$ is given by the empirical model found in [34], which was formulated by relating capacity and time in experiments where the discharge power P was hold constant:

(14) $$t=\delta \left(N\right)\cdot P^{\varepsilon \left(N\right)}\cdot \;{\hat{C}\left(t\right)}^{\beta },$$

where N is the number of cells in a series of the battery pack, while β>0, δ>0 and ε<0 are battery-dependent parameters to be determined experimentally. That model is appealing since it does not require knowledge of voltage variations during the discharge.

A more generalized form for (11) is used in this paper for variable power, that is:

(15) $$\hat{C}{\left(t\right)}^{\beta }=\int_{0}^{t}\frac{1}{\delta {P\left(t\right)}^{\epsilon \;}}dt.$$

Clearly, (11) and (12) coincide when P is constant. It can be observed that, for any time interval in which P is constant, the discharged capacity C is linear with respect to time when it is raised to the power of β, so that all the results are presented by considering ${C(t)}^{\beta }$. Moreover, several experimental tests to identify β showed that this parameter has small variations with the variation of the power. Given this small variation ($\sigma_{\beta }=53.4\times 10^{-3}$), one can assume β=0.9648 as a constant value equal to the mean of the evaluated β from the tests and focus on fitting the model on parameters δ and ε.

Initial guesses for parameters δ and ε, denoted as $\delta_{0}$ and $\varepsilon_{0}$, were found in the literature [34] as follows:

(16) $$\delta_{0}\left(N\right)=-0.1067{\cdot N}^{3}+0.8960\cdot N^{2}+2.488\cdot N+0.6299=18.089\;\frac{s^{1-\beta }}{W^{\epsilon }A^{\beta }\;},$$

and

(17) $$\varepsilon_{0}\left(N\right)=2.917\cdot 10^{-4}\cdot N^{3}-1.375\cdot 10^{-3}\cdot N^{2}+3.083\cdot 10^{-3}\cdot N-1.041=-1.032,$$

assuming that the number of cells is N=4. Measurement unit of $\delta_{0}$ is almost volt if $\epsilon_{0}\approx -1$ and β≈1.

Parameters estimation is performed with nonlinear curve-fitting using the least-squares method. We denote with index j the *j*-th experiment, which is a collection of measurements performed at instants $t_{j,i}$, where i is the *i*-th time sample, starting from time zero for each experiment. Measurements of current, power, and discharged capacity are denoted as $I_{j,i}$, $P_{j,i}$, and $C_{j,i}$, respectively. $C_{j,i}$ is calculated at instants $t_{j,i}$ by numeric integration of (10) using global adaptive quadrature, i.e., the ‘integral’ function in MATLAB, and it is predicted by model (12), which gives estimates ${\hat{C}}_{j,i}$. Experiments (and their indexes j) have been divided into two subsets of train T and validation V. 

Parameters δ and ε are then obtained by solving:

(18) $$\underset{\delta ,\;\epsilon \;}{min}\sum_{j\in T}\sum_{i}{\left({\hat{C}}_{j,i}^{\beta }-C_{j,i}^{\beta }\right)}^{2}.$$

To make a comparison of results obtained with different tests, residuals are calculated as follows:

(19) $$r_{j,i}=C_{j,i\;}-{\hat{C}}_{j,i}.$$

The residual can be interpreted as a time error if a conventional current of 1 A is assumed. If the time error is given in seconds and the residual in milliampere·hour, it is obtained as follows:

(20) $$\frac{\Delta t_{j,i\;}}{s}=3.6\times \frac{r_{j,i}}{mA\cdot h}.$$

## 4. Definition of Real-World Test Cases

To evaluate the limits and performance of the model described above, different experimental tests are performed by taking into account different operating conditions. To identify realistic power levels for testing the model, test cases related to fixed- and rotary-wing aircraft have been considered. 

Let us consider some real-world cases, in particular, a radio-controlled glider will be considered as a fixed-wing aircraft and a 7 inch quadcopter will be examined as rotary-wing aircraft.

Starting from the fixed-wing aircraft, the examined glider is the Phoenix S by Volantex RC [41], a 1.6 m wingspan glider. The measured battery power at the bench is 35 W at cruising speed (60 km/h) and 124 W when climbing at a rate of 8 m/s. The wing planform is $0.295{\;m}^{2}$ and the mass is 0.98 kg. Plugging the values in Equation (3) the mechanical powers for climbing, that is the power at the takeoff of the aircraft, and the mechanical power for cruising, that is the power required by the aircraft to fly in a straight line holding the altitude, are 54.8 W and 16.4 W, respectively. These mechanical powers translate to the power requested by the battery by the total efficiency factor $\eta_{tot}$, which is assumed to be equal to 0.5, so:

(21) Pbatt=PRηtot,

hence the power requested by the battery at the take-off is 109.6 W and the power requested by the battery for cruising is 32.8 W. The fixed-wing aircraft can be reproduced in a laboratory by a test using two powers, to simulate the take-off at high power and a flight at cruising speed.

As for the multirotor, let us consider a typical amateur 7 inch quadcopter with a mass m=0.6 kg that flies in a straight line at constant power and increases the power to climb and decreases the power to lose altitude. Moreover, let us consider a 7 × 3 × 2 (7 inch diameter, 3 inch pitch, bi-blade) propeller by APC [42] for which all the parameters that fully characterize the propeller are given. For what is the aim of this work, one can consider, the given thrust and power coefficients to be constant and equal to $C_{T}\approx 0.0823$ and $C_{P}\approx 0.0315$ without loss of accuracy, as long as the propeller speed does not change much, e.g., we will be considering speeds between 100 rev/s and 150 rev/s.

As for the simplified model (5), the total mechanical power required to hold the quadcopter at a fixed altitude can be found by looking for the thrust that equals its weight. Assuming that all four motors produce the same power so that the weight is equally distributed, one can isolate the required propeller speed from (7):

(22) Ωh=Th4CTρD4=mg4CTρD4=122 rev/s.

Hence, plugging (19) into (6), the required power for a single motor is 12.2 W. Therefore, considering the typical efficiency of a small electric BLDC motor for quadcopters of 0.9, the total efficiency needed to convert the mechanical power into the electrical power requested from the battery is η=0.65. Then, accounting for the total power required by the battery to cruise at slow speed, i.e., to hold the altitude:

(23) $$P_{batt,h}=4\cdot \frac{P_{motor,h}}{\eta }=75.2\;W,$$

where the constant 4 is required as at first a quarter of the mass of the quadcopter was considered.

It is then easy to calculate the power needed for the quadcopter to change altitude. Consider the case of the quadcopter climbing at a rate $\dot{h}=3\;m/s$, the contribution $W\dot{h}=mg\dot{h}=17.7\;W$ must be added to the requested power. The total power required by the battery will then be:

(24) $$P_{batt,climb\;3\;m/s}=\frac{4\cdot P_{motor,h}+W\dot{h}}{\eta }=102.4\;W.$$

Similarly, to climb at a speed of 6 m/s, the total power will be $P_{batt,climb\;6\;m/s}=129.5\;W$. In the case of the quadcopter descending at a ratio of $\dot{h}=-3\;m/s$, the total power should be reduced to $P_{batt,descend\;3\;m/s}=48.0\;W$.

The scenario of a multirotor can be reproduced in the laboratory using four levels of power applied for randomized times.

## 5. Discharge Tests 

To simulate realistic cases, the following experimental tests have been performed.

### 5.1. Constant Power Test

The first tests are performed at constant power for the estimation of a discharge pattern of the batteries. Four different tests were carried out, at powers of 50 W, 75 W, 100 W, and 125 W.

### 5.2. Two-Step Power Test

**Figure 6:**
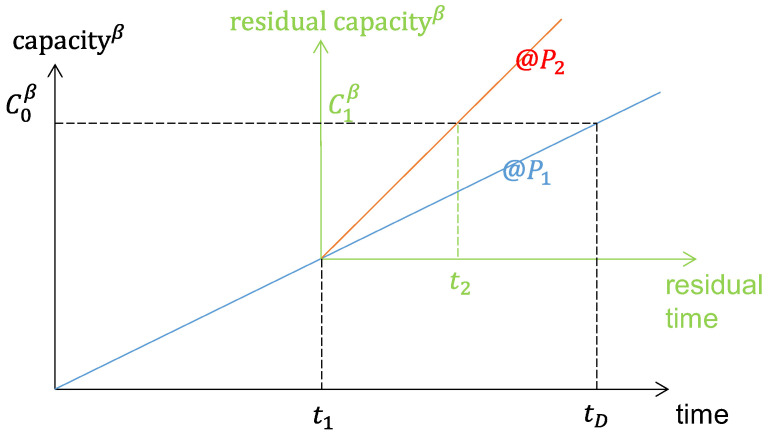
Two-step power test example.

Once the behavior of the discharge formula at constant power has been described, the next step is to evaluate the discharge model by considering two different power levels as shown in Figure 6, that would approximate the behavior of a fixed-wing aircraft, as explained above. 

The discharge can be modeled by applying (11) twice on constant power time intervals of duration $t_{1}$ and $t_{2}$, which are properly designed as follows. 

Considering (11), one can find the discharge time $t_{D}$ obtained when a constant power $P_{1}$ is applied:

(25) $$t_{D}=\delta \;P_{1}^{\epsilon }\;C_{0}^{\beta }.$$

After $t_{1}$, the residual capacity $C_{1}$ can be calculated from:

(26) $$t_{D}-t_{1}=\delta \;P_{1}^{\epsilon }\;C_{1}^{\beta }.$$

When a different power value $P_{2}$ is applied at $t_{1}$, it is assumed that the residual time to discharge can be calculated by using (11) again on the residual capacity, i.e., $t_{2}=\delta P_{2}^{\epsilon }\cdot C_{1}^{\beta }$, and substituting (22) and (23), the following is obtained:

(27) $$t_{2}=\delta \;P_{2}^{\epsilon }\;C_{0}^{\beta }-{\left(\frac{P_{2}}{P_{1}}\right)}^{\epsilon \;}\cdot t_{1}.$$

Experiments can be designed by calculating, for any chosen $P_{1}$ and $P_{2}$, durations $t_{1}$ and $t_{2}$ that discharge the battery capacity $C_{0}$. By letting, without loss of generality $t_{2}=k\cdot t_{1}$, it follows that the total discharge time is $t_{test}=t_{1}+k\cdot t_{1}$ and

(28) $$t_{1}=\frac{\delta \;P_{2}^{\epsilon }\;{C_{0}}^{b}}{k+{\left(\frac{P_{2}}{P_{1}}\right)}^{\varepsilon }}.$$

The tests were performed at 75 W and 100 W, as these are the power levels used in the previous constant power tests. Furthermore, the tests were carried out first using $P_{1}=75\;W$ and $P_{2}=100\;W$, then another set of tests was performed swapping $P_{1}$ and $P_{2}$ values. Three different values of k were chosen, corresponding to different ratios between duration at $P_{1}$ and $P_{2}$, hence a total of six tests were performed, each one characterized by different times and power levels. The values of k were:

- k=1: durations at ending and starting power are equal;
- k=2: duration at final power is twice the duration at starting power;
- k=0.5: duration at final power is half the duration at starting power.

The list of two-step experiments and their duration is shown in Table 3. 

### 5.3. Test with Amplitude-Modulated Pseudo Random Binary Signal (APRBS)

In order to model a more complex battery usage scenario, the model used is the more general one, that is the one described by (12).

To validate this model, it has been decided to perform a series of tests using pseudo-random signals. In this way, the model can be validated on a larger set of power levels, each of them applied at a random time. Four different power levels are applied to the battery for a quadcopter holding its altitude or climbing or descending at a constant rate, as was described in Section 4.

**Figure 7:**
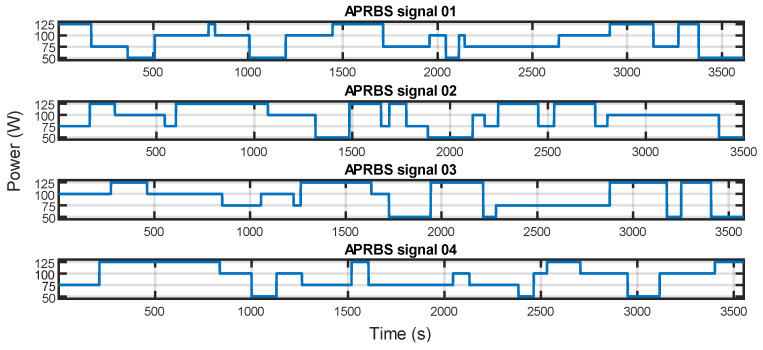
APRBS signals used for the tests.

Each power level of the test sequence was chosen randomly among the same values used in previous tests, i.e., 50 W, 75 W, 100 W, and 125 W. Each power was kept constant for a randomly selected time duration with uniform distribution over the interval [30, 300] s. The minimum total duration of the discharge test was set to 3500 s. Four tests were performed, resulting in mean time-averaged powers of 89.4 W, 97.3 W, 95.7 W, and 93.9 W. The signals used for the experiments are showed in Figure 7.

## 6. Results and Discussion

Table 4 indicates the training and validation sets used for the three different kinds of experiments. For the constant power tests, the highest and the lowest powers are used for the training in order to predict the behavior at intermediate powers. The same is carried out for the APRBS tests: the experiments that presented the maximum and the minimum mean power are used for the training. For the two-step tests, one test for each k-value is used for the training.

Training has provided the model coefficients listed in Table 5 that are used to evaluate the performance of discharge estimation for different tests. For reference purposes, the coefficients calculated in the literature (Equations (7) and (8) of [34]) are reported too. 

**Figure 8:**
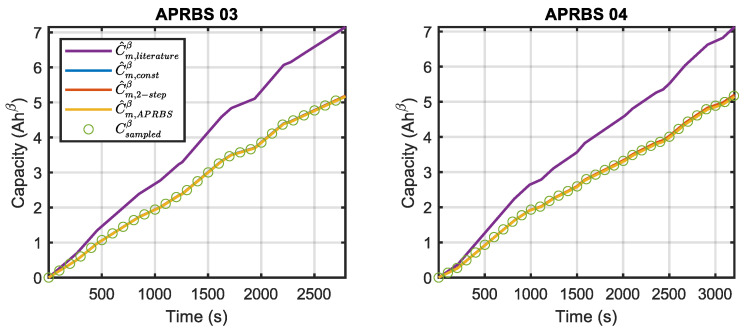
Validation for APRBS discharges.

For each test, the discharged capacity prediction is obtained by using (12), the measured power, and the different estimated models. As an example, the outputs of the models considered for validation of APBRS discharges are provided in Figure 8. To better represent model (12), measured capacities ($C_{sampled}$) and predicted ones ($\hat{C}$) are raised to the power of β. It can be clearly seen that all the models fitted in this work overlap with the sampled data; obviously, the model based on the literature parameters produces different results since it was obtained for different battery models. In the following, only the novel fitted parameters will be considered.

### 6.1. Qualitative Evaluation of Prediction Errors

**Figure 9:**
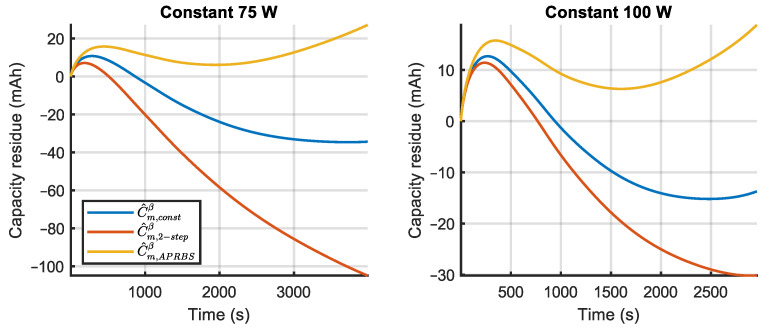
Prediction error of the three estimated models for constant power validation experiments.

**Figure 10:**
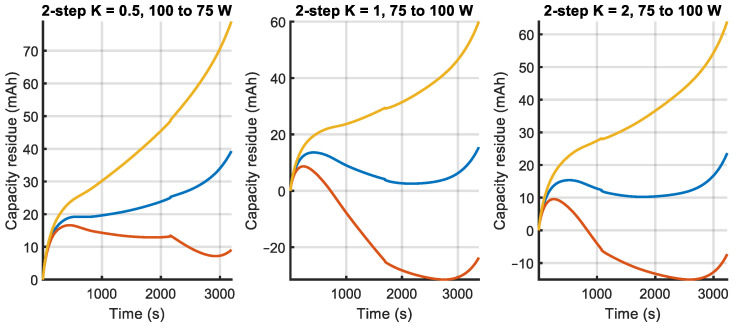
Prediction error of the three estimated models for two-step power validation experiments (legend is given in Figure 9).

**Figure 11:**
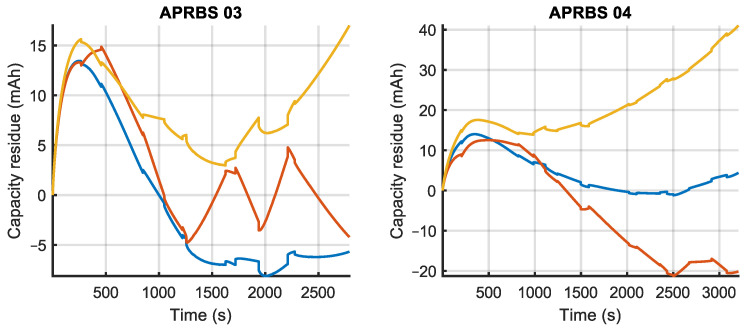
Prediction error of the three estimated models for APRBS power validation experiments (legend is given in Figure 9).

The prediction errors for the validation experiments, calculated by (16), are shown in Figure 9, Figure 10 and Figure 11, where the different estimated models are compared. It can be observed that, for the constant power tests, errors in model $m_{const}$ are bounded by the other two models. This happens also for the two-step tests and, to a lesser degree, for the APRBS tests. In many tests, model $m_{APRBS}$ appears to diverge faster than the others, giving larger errors at the end of the experiment; it performs worst especially in the two-step test, with an error that increases monotonically. To better compare the performance of the models and discriminate between the behavior of $m_{const}$ and $m_{2-step}$, further analysis is carried out using Root Mean Square Error (RMSE) and final error.

### 6.2. Comparison of RMSE and Final Error

To carry out a comparison the RMSE is calculated for all samples $N_{j}$ for each experiment j:

(29) $$RMSE_{j}=\sqrt{\frac{\sum_{i=0}^{N_{j}}{\left(r_{j,i}\right)}^{2}\;}{N_{j}}}.$$

**Figure 12:**
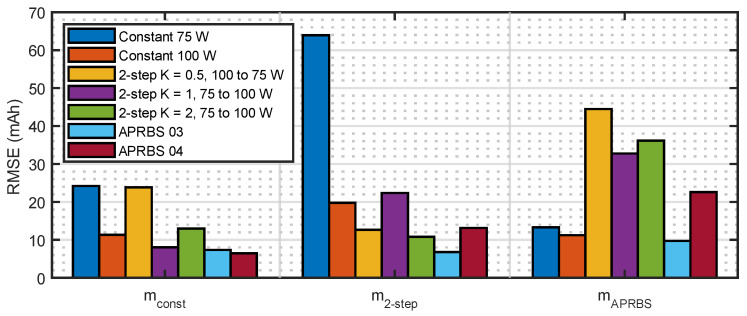
Root Mean Square Error of capacity prediction. Series are grouped by estimated model.

The bar plot in Figure 12 shows three different series, grouped by estimated model. 

Let us consider the first series, that is, all the predictions of the model $m_{const}$. It can be seen that fitting the parameters on the constant power experiments provides a model that is as good, or even better, also when two-step and APRBS discharges are to be predicted since the RMSE over the tests can be lower. This is indeed good news for the methodology: performing constant power tests for the characterization of the battery is easier and does not need a complex signal such as the ones used in the APRBS tests, so there is less chance of errors during the procedure.

Surprisingly, when considering the last series, that is, the one related to model $m_{APRBS}$, it happens that predictions are better overall, in terms of RMSE, when constant power discharges are to be predicted. However, that prediction case is not really useful. Since it is a real-world scenario, the demand for power of an electrical brushless motor on a UAV is not really constant but has several different variations. Therefore, the characterization of the battery pack by estimating $m_{APRBS}$, would be more difficult without gaining any advantage with respect to the easier test at constant power.

The second series, that is, the one that uses model $m_{2-steps}$, performs better for APRBS tests with respect to constant power tests, similar to model $m_{const}$ shown in the first series but, in comparison to $m_{const}$, it has a worse RMSE overall. Hence, model $m_{const}$ is preferable to model $m_{2-steps}$.

If the results of Figure 12 are grouped by discharge type of the validation experiment, it is found that two-step and APRBS discharges are better predicted by model $m_{const}$ compared to the other models. Instead, constant discharges are better predicted by $m_{APRBS}$; however, this case is of lesser significance, as already mentioned. 

**Figure 13:**
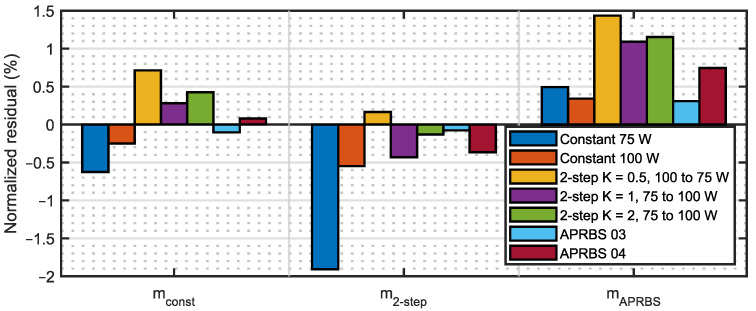
Normalized prediction error at the end of discharge. Series are grouped by model.

Finally, the capacity prediction error at the end of different discharges is compared. For that purpose, it is useful to normalize this feature on the full scale of the test:

(30) $$R_{j,final}=\frac{r_{j,final}}{C_{j,final}}\times 100\%,$$

and the results can be represented in a bar plot as shown in Figure 13.

The results are very similar to the ones already described for the RMSE: both $m_{const}$ and $m_{2-step}$ perform better than $m_{APRBS}$ set.

It can be concluded that the non-optimal results obtained by using model parameters fitted on APRBS tests suggest that increasing the number of power level shifts does not necessarily lead to a better model. Hence, it is convenient to use constant power tests to characterize the battery pack.

## 7. Conclusions

In this paper, the author presented a methodology for evaluating the discharged capacity of a Li-Po battery that would complement other studies on the evaluation of the SOH of the same battery. This methodology is investigated in such a way that users could perform easy experiments on their own batteries. In particular, it has put a heavy focus on looking for a methodology that would use simple signals for the characterization of a battery pack. Moreover, the model used for identification is required to be simple and not computation-heavy. The resulting one is an integral model with only three easy-tunable parameters.

The results show that performing experiments using constant power levels corresponding to the expected maximum and minimum powers provides enough data to fit the parameters of the proposed model, supporting interpolation for all the other power levels. These parameters allow any user to reconstruct the discharge curve of a battery using a model that requires supplied power as the only input.

Performing tests at constant powers not only provides data for predictions relevant to that kind of discharge, but power can follow ideally other time dependencies, as shown in the APRBS validation experiments. Indeed, results in terms of RMSE and residual at the end of the test showed that, for time-varying discharge powers, models estimated at constant power may actually outperform models estimated with APRBS tests.

Using this methodology, the final user can accurately choose the battery for the task, minimizing any risk of miscalculating the energy required for the task itself, which would lead to mission failure or inefficiency.

However, the technique used does not take into account changes in the operating temperature of the battery or its aging, nor the room temperature. It is well known in the literature that these variables affect the performance of a LiPo battery, but the proposed methodology is aimed at finding an easy-to-use model, with few parameters without high computational cost. Nevertheless, these effects will be further analyzed and techniques of compensation for these factors will be considered in an upcoming work.

## Figures and Tables

**Table 1:** Current values used for the amended VDA test procedure.

Duration (s)	Applied Current	Phase
100	0	Relaxation
400	I	Discharge
200	0	Relaxation
350	−I	Charge
100	0	Relaxation

**Table 2:** Calculated resistances from current-off tests.

Test Current (A)	Resistance (mΩ)
2	20.48
4	21.24
6	20.46
8	20.51

**Table 3:** Two-step power tests.

nr.	*k*	*P*_1_ (W)	*P*_2_ (W)	*t*_1_ (s)	*t*_2_ (s)	*t*_test_ (s)
1	1	75	100	1708.5	1708.5	3417.0
2	1	100	75	1708.5	1708.5	3417.0
3	2	75	100	1086.7	2173.5	3260.2
4	2	100	75	1196.5	2393.1	3589.6
5	0.5	75	100	2393.1	1196.5	3589.6
6	0.5	100	75	2173.5	1086.8	3260.3

**Table 4:** Training and validation sets.

Experiment Type	Training	Validation
Constant	50 W and 125 W	75 W and 100 W
2-step	k = 0.5, 75 W to 100 W k = 1, 100 W to 75 W k = 2, 100 W to 75 W	k = 0.5, 100 W to 75 W k = 1, 75 W to 100 W k = 2, 75 W to 100 W
APRBS	Signal 1 and signal 2	Signal 3 and signal 4

**Table 5:** Models estimated for different training experiments.

Test	Model Name	δ	ε	β
Literature	$m_{literature}$	18.0891	−1.0320	0.9664
Constant	$m_{const}$	23.6482	−1.0209	0.9648
2-step	$m_{2-step}$	20.3266	−0.9886	0.9648
APRBS	$m_{APRBS}$	23.4535	−1.0192	0.9648

## Data Availability

The data presented in this study are available on request from the corresponding author.
